# Efficiency of Base Excision Repair of Oxidative DNA Damage and Its Impact on the Risk of Colorectal Cancer in the Polish Population

**DOI:** 10.1155/2016/3125989

**Published:** 2015-11-16

**Authors:** J. Kabzinski, B. Mucha, M. Cuchra, L. Markiewicz, K. Przybylowska, A. Dziki, L. Dziki, I. Majsterek

**Affiliations:** ^1^Department of Clinical Chemistry and Biochemistry, Medical University of Lodz, Plac Hallera 1, 90-647 Lodz, Poland; ^2^Department of General and Colorectal Surgery, Medical University of Lodz, Plac Hallera 1, 90-647 Lodz, Poland

## Abstract

DNA oxidative lesions are widely considered as a potential risk factor for colorectal cancer development. The aim of this work was to determine the role of the efficiency of base excision repair, both in lymphocytes and in epithelial tissue, in patients with CRC and healthy subjects. SNPs were identified within genes responsible for steps following glycosylase action in BER, and patients and healthy subjects were genotyped. A radioisotopic BER assay was used for assessing repair efficiency and TaqMan for genotyping. Decreased BER activity was observed in lymphocyte extract from CRC patients and in cancer tissue extract, compared to healthy subjects. In addition, polymorphisms of *EXO1*, *LIG3*, and *PolB* may modulate the risk of colorectal cancer by decreasing (*PolB*) or increasing (*LIG3* and *EXO1*) the chance of malignant transformation.

## 1. Introduction

Colorectal cancer (CRC) is a common neoplasia in both men and women and is ranked as the second most common type of cancer. The causes of colorectal cancer have not yet been established and its incidence is known to be increasing, with approximately 1.4 million new cases diagnosed each year [1]. CRC occurs mainly in three specific forms: sporadic form, which accounts for about 80% of all cases, a familial form, which represents about 15%, and inherited forms, observed in 5% of all cases, which include familial adenomatous polyposis (FAP), and hereditary nonpolyposis colorectal cancer (HNPCC) [2]. Despite the cause of most colorectal cancers being environmental factors, studies show that individual predispositions for developing this cancer may depend on mutations of certain genes, including those involved in the process of DNA repair. Several DNA repair mechanisms have evolved to protect the genome from DNA damage caused by endogenous or environmental factors, which if unrepaired could lead to the initiation of carcinogenesis. The efficiency of DNA repair varies between individuals, and the reasons for this should be sought in polymorphisms within the DNA repair genes. An increasing number of DNA repair gene polymorphisms are being correlated with increased risk of cancer occurrence. Although an irrefutable link has already been established between colorectal cancer and the presence of mutations in mismatch repair (MMR) genes [3], other polymorphisms of DNA repair genes (BER and NER) are undergoing investigation for a potential influence on CRC.

Base excision repair (BER) is DNA repair system that operates on small lesions such as oxidized or reduced bases. A single damaged base is removed by base-specific DNA glycosylases. The abasic site is then rebuilt by endonuclease action, removal of the sugar residue, DNA synthesis using the other strand as a template, and then ligation. The molecules involved with the process include Exonuclease 1, which cleaves the nucleotides from the end of DNA strand, DNA polymerase beta, which is involved in gap filling, and DNA ligase 3, which seals interruptions in the phosphodiester backbone of duplex DNA [4]. Polymorphisms in the genes encoding these proteins are suspected to influence the efficiency of the whole BER process and thus modulate the risk of CRC.

The present study has two major aims. The first is to evaluate the influence of the presence of polymorphisms within the tested genes with an elevated risk of colorectal cancer. The second is to make an* in vitro* assessment of the efficiency of restoring DNA continuity via the BER pathway. As all protein products of the genes chosen for SNP screening are involved in the BER stages directly following glycosylase action, the BER assay was adjusted to measure only the gap-filling step, when* PolB*,* EXO3,* and* LIG3* play crucial roles.

## 2. Materials and Methods

DNA for genotyping was isolated from lymphocytes of the peripheral blood. The blood samples were taken from 235 unrelated patients hospitalized in the Military Medical Academy University Teaching Hospital-Central Veterans' Hospital in Lodz. Each patient had histopathologically confirmed colorectal cancer. The studied group included 137 men and 98 women (average age 61 years ± 8 years). The stage of the tumors was established according to TNM scale. The control group included 240 individuals not diagnosed with cancer and with ages corresponding to the age of the studied group (*p* < 0.05). Permission to conduct research was granted by the bioethics committee of the Medical University of Lodz.

DNA isolation was carried out with a commercial kit QIAamp DNA Blood Mini Kit for isolation of high-molecular-weight DNA (Qiagen).

The occurrence of polymorphic variants of 242Pro/Arg of* PolB* gene, 780Arg/His of* LIG3* gene, and 589Glu/Lis of* EXO1* gene was studied with TaqMan technique. Briefly, 25 *μ*L of reaction mixture was used for analysis, containing 1 *μ*L of genomic DNA solution, 1 *μ*L of probes designed specifically for each polymorphism, 13 *μ*L of premix with polymerase, and 10 *μ*L of water. The PCR reaction was performed in a Stratagene Mx3005P Real Time PCR Thermocycler. The RS numbers for polymorphisms and thermal conditions of reaction are shown in Table 1. For 10% of the randomly selected samples, genotyping was repeated to confirm reproducibility. Cases and controls were genotyped randomly and researchers were blinded to the case/control status during genotyping.

**Figure 1:**
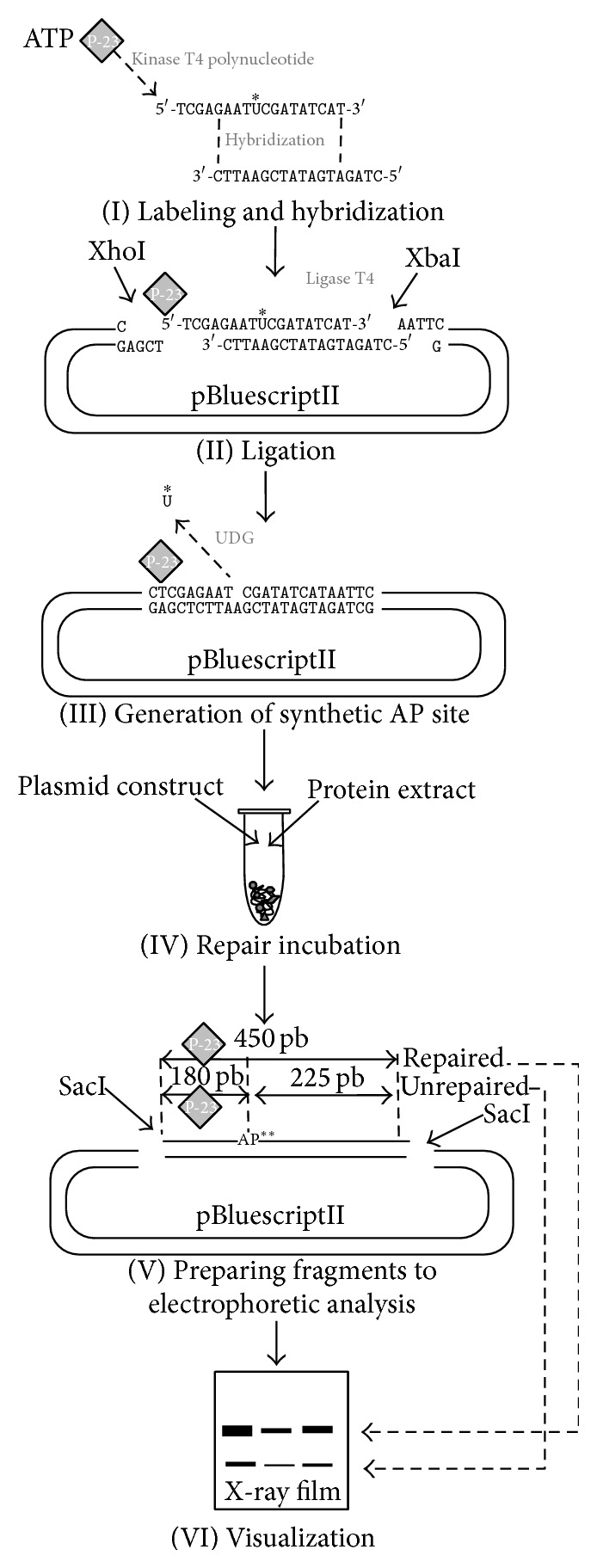
(I) A uracil-containing oligonucleotide was subjected to action of polynucleotide kinase to attach a radioactive phosphate group from [*γ*-^32^P] ATP. It was hybridized with a second oligonucleotide whose sequence was adjusted to obtain sticky ends referring to XhoI and XbaI digestion site. (II) The short DNA fragment prepared in stage I was cloned into a pBluescriptII plasmid. (III) Uracil-DNA glycosylase was utilized to remove uracil and, as consequence, create a single gap in DNA to act as a synthetic lesion. (IV) A plasmid with single AP site constituted a substrate for the protein extract in 90-minute repair incubation. (V) Two SacI recognition sites of the pBluescriptII plasmid were used to excise 450 pb-long fragment covering the lesion site and radioactive label for analysis on 8% urea/acrylamide gel. (VI) Interpretation of outcomes was based on detection of two bands. The full-length 450 pb fragment reflects restored DNA fraction, whereas presence of short 180 pb fraction indicates the amount of unrepaired DNA. ^*∗*^U: uracil; ^*∗∗*^AP: apurinic/apyrimidinic.

BER efficiency was evaluated according to Matsumoto et al. [5] with some minor modifications to improve the preparation of synthetic lesion site. A plasmid construct with radioactively labeled single-strand breaks was incubated with protein extract isolated from peripheral blood lymphocytes and slices of cancerous tissue removed during surgical procedures. Repair capability was assessed by densitometric analysis of DNA fragments which had been electrophoretically separated and depicted on X-ray film: these fragments vary with regard to length and can identify repaired or unrepaired fractions. The course of procedure is outlined in Figure 1.

The blood donors were a 79-year-old man with histopathologically confirmed adenocarcinoma and a cancer-free woman of the same age. A colorectal cancer tumor had been removed from the 86-year-old female with cecum carcinoma. Control tissue samples of the colon were taken from patients with primary inguinal incarcerate hernia from the macroscopically unchanged tissue during the operation. All individuals enrolled in this experiment were hospitalized in the Military Medical Academy University Teaching Hospital-Central Veterans' Hospital, Lodz.

### 2.1. Details of the BER Assay Procedure

#### 2.1.1. Preparation of DNA Substrate

The Vector, a pBSII plasmid, was multiplied in* E. coli* DH5*α* and isolated by Qiagen Maxiprep and underwent double digestion with 2.5 U of XbaI and 2.5 U XhoII fast digest enzymes for 1 hour (Fast digest, ThermoScientific, Rochester, USA). SAP (ThermoScientific, Rochester, USA) enzyme was applied to avoid self-ligation. Insert 5′-TCGAGAATUCGATATCAT-3′ was labeled in kinase reaction 2 U T4 kinase polynucleotide (thermo) with 2 *μ*L of [*γ*-^32^P] ATP (6000 *μ*Ci) whereas the second oligo (5′-CTTAAGCTATAGTAGGATC-3′) was incubated under the same conditions but with unlabeled ATP. Equal amounts of the two oligonucleotides were mixed and annealed through heating to 95°C before being left to slowly cool down. A 1 : 5 vector : insert molar ratio (established previously) was applied to allow ligation with 1 U of T4 ligase (ThermoScientific, Rochester, USA) overnight. The construct was purified by elution (GenJet Maxi prep kit, ThermoScientific) from 1% agarose gel.

Preparation of whole-cell protein extract is as follows: Minute Total Protein Extraction Kit (Invent) was used to isolate proteins from peripheral blood lymphocytes or tissue slices. All protein samples were adjusted to 2 *μ*g/mL.

#### 2.1.2. Repair Assay

A 100 ng/reaction of unaltered native pBSII (load control) was mixed with prelabeled plasmid (1000 cpm/sample) carrying an AP site generated by digestion with 1 U of UDG glycosylase (ThermoScientific) for 4 h. This mixture was made up to a total volume of 15 *μ*L by adding the reagents 0.4 *μ*L of 1 M HEPES-KOH, pH 7.5, 0.2 *μ*L of 1 M MgCl_2_, 0.5 *μ*L of 3 M KCl, 0.2 *μ*L of 0.1 M DTT, 1 *μ*L of 0.1 M ATP, and 0.4 *μ*L of 1 mM dNTP and adjusted to a final volume with H_2_O. The reaction was launched by adding 5 *μ*L (10 *μ*g) protein extract. Repair incubation was carried out in a thermocycler (Bio-Rad) and took 90 min at 25°C. The reaction was stopped by adding of 6 *μ*L of 2% SDS to each tube. Thereupon, 2 *μ*L of 1 mg/mL proteinase K and 2 *μ*L of 0.2 mg/mL carrier tRNA were added to each tube and incubated at 37°C for 30 min, and then treated with 150 *μ*L stop solution (10 mM Tris-HCl, pH 7.5, 300 mM sodium acetate, 10 mM EDTA pH 8.0, 0.5% SDS). DNA was recovered by phenol/chloroform extraction (1 : 1) and overnight ethanol precipitation. A 450 pb length DNA fragment was excised from plasmid by 1 U of SacI enzyme (ThermoScientific, Rochester, USA) incubated at 37°C for 1 h. To allow the repaired and unrepaired fractions to be differentiated, the remaining unrepaired AP site was treated with 1 U of AP-recognizing endonuclease IV for 1 h. All samples were run on 8% urea-containing polyacrylamide gel for 3 h in 120 V. The accurate electrophoresis was preceded by 1 h preelectrophoresis with loading buffer.

#### 2.1.3. Visualization

The bands were detected by autoradiography. The gels were dried and stored at −20°C with X-ray film for 2 h, 6 h, or overnight exposure. Bromidium ethidium staining was used to visualize load control. Optical density quantification of bends was performed with GeneTools software (Invitrogen).

## 3. Results

The genotyping results indicate that the Lys/Glu genotype of the* EXO1* gene (Table 2) may increase the risk of colorectal cancer (OR = 1.672 (1.109–2.519), *p* = 0.014). The investigated* PolB* gene polymorphism was not found to increase the risk of CRC; however, our analysis suggests that occurrence of Arg allele may have a protective effect, since it decreases the risk of colorectal cancer (OR = 0.772 (0.601–0.994), *p* = 0.044) as shown in Table 3. The 780Arg/His polymorphism of the* LIG3* gene was found to contribute to an increase in the risk of CRC (OR = 1.570 (1.109–2.224), *p* = 0.011) (Table 4).

In order to investigate the interaction of the polymorphisms of the studied genes and to evaluate their mutual influence on the risk of colorectal cancer, gene-gene interactions were analyzed. The simultaneous occurrence of the Lys/Glu genotype of the* EXO1* gene and the Pro/Pro genotype of the* PolB* gene was found to possibly increase the risk of colorectal cancer (OR = 2.265 (1.193–4.301), *p* = 0.011) (Table 5). In case of gene-gene interactions between 589Lys/Glu* EXO1* SNP and 780Arg/His* LIG3* SNP, the simultaneous occurrence of Lys/Glu and Arg/His genotypes may increase risk of colorectal cancer (OR = 1.970 (1.041–3.731), *p* = 0.036) while concomitant presence of Glu/Glu and Arg/Arg genotypes may decrease the risk (OR = 0.402 (0.178–0.906), *p* = 0.026) (Table 6). Finally, the analysis of gene-gene interactions for 242Pro/Arg* PolB* gene and 780Arg/His* LIG3* gene indicated that the cooccurrence of genotypes Pro/Pro and Arg/His may increase the risk of CRC (OR = 2.154 (1.265–3.667), *p* = 0.004) (Table 7).

**Figure 2:**
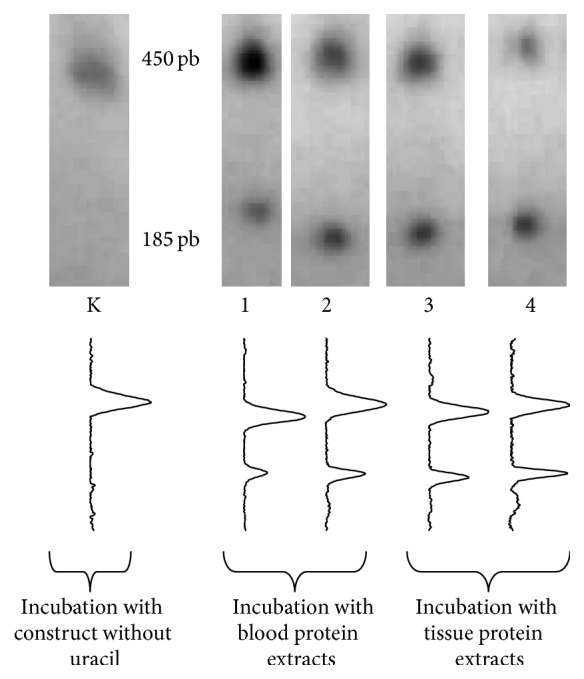
A comparison of BER activity in the lymphocytes and tissue of CRC patients and healthy controls. Each electropherogram shows two fractions of DNA: 450 pb repaired and 185 pb unrepaired. Lanes 1-2 indicate lymphocyte BER efficiency while lanes 3-4 refer to BER in tissue. Samples are presented in the following order: K: positive control; DNA substrate did not contain uracil and so reflects 100% of repair; 1: healthy control; 2: colorectal cancer; 3: unchanged colon tissue; 4: colorectal cancer.

In general, optical density detection of particular DNA bands revealed higher BER repair efficiency among cancer-free individuals than CRC patients in both lymphocytes and colon tissue samples. The percentage ratio of repaired to damaged fractions was found to be 89.67%/10.32% in lymphocytes taken from healthy subjects and 70.5%/29.5% in those of CRC patients. Examination of the ability of tissue protein extract to perform BER indicated a significantly greater repair level in normal tissue (68.11%/31.89%) than CRC tissue (58.36%/41.64%). The results of the BER assay analysis are presented in Figure 2.

## 4. Discussion

All cells in the human body are permanently exposed to the negative effects of reactive oxygen species. Virtually all kinds of cell components, including proteins, lipids, and nucleic acids, can be targets for attack by ROS, which may interfere with the proper functioning of cellular biochemical processes. Oxidative stress has been confirmed to play a role in carcinogenesis by a number of previous studies [6]. Oxidative damage to DNA has significant mutagenic potential, and excessive accumulation of damage to DNA leads to cell necrosis or apoptosis, the most abundant types of lesion being 2,6-diamino-4-hydroxy-5-formamidopyrimidine (FapyG) and 4,6-diamino-5-formamidopyrimidine (FapyA) and 8-oxo-7,8-dihydroguanine (8-oxoG).

By virtue of its high stability and relative simplicity of detection, 8-oxoG is a vital biomarker of DNA oxidative damage [7]. Hence, colorectal cancer was extensively examined in terms of 8-oxoG presence. An analysis performed on a Spanish population indicated a twofold higher level of 8-oxoG in colorectal tumors than in normal mucosa [8]. In other studies, immunohistochemical tests complemented by high-performance liquid chromatography (HPLC) have revealed considerable higher levels of 8-oxoG in colorectal carcinoma than nontumorous colon epithelial cells [9]. Furthermore, the same team reports the presence of an elevated 8-oxoG level accompanied by 8-oxoG lyase overexpression [10]. Increased levels of 8-oxoG have been observed in CRC patient lymphocytes [11] and plasma [12] compared to those of healthy controls.

These high levels create a very challenging environment for base excision repair processes, which have to operate with high efficiency to maintain genome integrity. Omission of oxidized forms of guanine may lead to incorrect base-pairing, resulting in G:C to T:A transversion mutation [13, 14]. However, no evaluation of BER system activity can be performed purely on the basis of 8-oxoG level. In addition, the majority of previous studies tests have been based on the total 8-oxoG content including unbound 8-oxoG and that bound to DNA. A high level of free 8-oxoG might occur as consequence of efficient performance of the BER initial step, when the damaged bases are being recognized and excised by glycosylase. Conversely, higher numbers of damaged bases remaining in a DNA-associated form may indicate that BER activity is insufficient to cope with repairs.

A more precise tool to evaluate the level of DNA single-strand breaks and repair capacity is the comet assay. An alkaline version of the comet assay used in a previous investigation indicated statistically significant differences in repair efficiency between CRC patients and healthy subjects. After a 240-minute repair incubation, the level of single-strand DNA breaks was significantly diminished in lymphocytes from a cancer-free control group in comparison to CRC subjects. A similar difference was observed in a comparative analysis of cells from normal colon mucosa tissue and a CRC tumor [15]. In the course of a comet assay, cells are incubated intravitally after hydrogen peroxide treatment, whereby the BER process, consisting of the excision of the damaged base and restoration of the DNA sequence, can be tracked holistically.

Glycosylase activity in initial stages of BER is an issue that has been the focus of a great degree of research interest. Thus far, eukaryotic cells have been found to possess several glycosylases such as NEIL1-3, UNG, NTH1, MUTYH, APE1, and OGG1 [16–19]. OGG1 is the primary BER enzyme capable of cleaving *N*-glycosyl bond between the sugar component and 8-oxoG. Studies based on a mouse model with* OGG1*−/− knock-out revealed this deficiency to have minor or even marginal importance in pathogenesis and cancer frequency [20]. MUTYH has a unique ability to remove normal adenines misincorporated opposite to 8-oxoG. Similar to* OGG1*, studies based on biallelic* MUTYH* mutation implied no significant increase in sensitivity to oxidative stress [21]. Surprisingly, an additive effect has been observed in mice with the double mutation* OGG1*−/− and* MUTYH*−/−, where higher tumor appearance frequencies have been noted [22].

Both* OGG1* and* MUTYH* have numerous polymorphic variants which are being eagerly examined in the context of carcinogenesis. The common polymorphisms of the* OGG1* gene, S326 C and R46Q, have been found to slightly decrease the activity of the enzyme [23, 24]. Regarding population screening, definitely more attention has been paid to the screening of S326 C, especially its involvement in lung cancer development. However, several investigations summarized in a meta-analysis do not reveal any linkage with lung cancer [25]. In contrast, certain variants of* MUTYH*, a polymorphism-rich gene, have been shown to elevate the risk of CRC 28-fold [26]. A great deal of current research into genetic variation of proteins has focused on the* XRCC1* gene. XRCC1 is an important protein due to its participation in the recruitment of the other BER proteins, making it a binder of all stages [27]. However, several large scale meta-analyses display contradictory conclusion about its role in carcinogenesis. To be specific, while the Arg194Trp polymorphism was found to have a protective effect on tobacco smoking with regard to cancer risk [28], it was found to have no such role for other examined cancers [25, 29, 30].

As the literature shows no consensus on role of the early stages of BER, the present study addresses the gap-filling stage. The present study is so far the only one aimed to evaluate the effectiveness of BER in CRC. Undoubtedly, although our findings show an interesting trend, they should be treated with great caution. As a BER deficiency can be observed in lymphocytes from a CRC individual, it can be inferred that the native repair system is also deficient, which may result in a slow, gradual accumulation of damage that, at some critical moment, may contribute to the development of cancer.

It is important to determine whether some difficult to exclude factors can interfere with the result. To minimize this risk of appearance of additional undesirable damaging agents, the primary inclusion criteria for the BER assay were place of residence (the same city), the subject not taking medication, including cancer therapy for CRC, and the lack of any smoking addiction or alcohol abuse. However, it is difficult to predict the influence of other significant factors such as ionizing radiation, UV light, diet, or stress associated with everyday situations. There is some risk that any of these factors could put BER on standby, while it is forced to repair more cellular proteins which had been produced as a response to greater exposure [31]. In the follow-up phase of the experiment, the tumor cell extract demonstrated a similar reduction of BER activity in comparison to normal tissue.

However, it is unclear whether this reduced repair ability is innate and this phenotype is maintained after tumorigenesis, as differences could emerge due to the presence of mutations which were nested during malignant transformation. In addition, weak BER capacity may help exacerbate the genotoxic effect of ROS, allowing malignancy to progress. Chan et al. report that the presence of hypoxia in CRC provokes changes in BER [32]. Other reports note that some characteristics of colon tissue factor may induce oxidative stress: an increased amount of free radicals may occur as result of diet rich in red meat [33] or with low calcium or vitamin D levels [34]. What is more, bacteria living in the intestine can also be an important extracellular source of ROS which promotes increased DNA damage in colonic epithelial cells [35]. The available evidence seems to suggest that BER has a possible impact on both the development and progression of CRC.

A similar concept has recently been presented by Stanczyk et al., who, by using a similar methodology to the present study, report significantly lower BER efficiency in lymphocytes taken from children suffering from childhood acute lymphoblastic leukemia in comparison to healthy controls [36]. Although CRC and leukemia are virtually incomparable due to their totally different natures, the BER system was found to play a crucial role in both and may also be involved in the pathogenesis of several other diseases.

It is undeniable that polymorphisms of DNA repair systems participate in the carcinogenesis process, as mentioned before in the first part of Section 4. Extensive studies suggest that these polymorphisms play a role in almost all types of cancer [37–40], including colorectal cancer [29, 41]. The genetic polymorphisms of MMR system appear to participate in the pathogenesis of hereditary nonpolyposis colorectal cancer [42, 43], and a growing body of evidence suggests their involvement in the BER system [44, 45], but reports concerning the NER system are inconclusive, with some confirming the link [46] and others denying it [47].

The present paper examines the impact of polymorphisms of* PolB*,* LIG3,* and* EXO1* of the BER repair system on the modulation of the risk of colon cancer. The genes were selected on the basis that the products of these three genes do not have glycosylase activity, thus avoiding any negative impact on the first part of the experiment. All three proteins are involved in stages of BER directly following the glycosylase action. Therefore, BER assay was adjusted to measure only the gap-filling step where* PolB*,* EXO1,* and* LIG3* play crucial roles. The protective effect of the Arg allele for the 242Pro/Arg gene polymorphism of* PolB* demonstrated in our work (Table 3) has been shown in previous publications [46]. In addition, the 242Pro/Pro genotype of the* PolB* gene in combination with the genotype 780Arg/His of* LIG3* gene increases the risk of CRC (Table 7), and the risk is much higher than in case of the 780Arg/His SNP of* LIG3* (OR = 2.154; 1.265–3.667, *p* = 0.004 versus OR = 1.570; 1.109–2.224, *p* = 0.011) (Table 4). This clearly shows the important role of gene-gene interactions in modulating the risk of malignant transformation, which has been confirmed in many other publications [48, 49].

Our finding that the 589Lys/Glu SNP of* EXO1* is associated with an increased risk of CRC is contrary to those of Akbari et al. [50]. However, it should be noted that the previous study was performed on an Iranian population, while our tests were carried out on a Polish population. Ethnic group has been repeatedly demonstrated to have a significant impact on the modulation of the risk of particular diseases [51, 52]. Yamamoto et al. [53] suggest that the potential impact of polymorphism 589Lys/Glu on increased risk of carcinogenesis may depend on the presence of cigarette smoking by the patient. Again, however, these studies concern a Japanese population, which may exert an influence on the results.

In our opinion, it is important to note interaction of polymorphisms 589Lys/Glu of the* EXO1* gene and 780Arg/His of the* LIG3* gene (Table 6), whose coexistence increases the risk of CRC compared to the presence of polymorphism 589Lys/Glu itself (OR = 1.970; 1.041–3.731, *p* = 0.036 versus OR = 1.570; 1.109–2.224, *p* = 0.011). As no extant publications describe the influence of the 780Arg/His polymorphism of* LIG3* on the risk of CRC, our own findings in this regard showing an elevated risk (Table 4) are significant. In addition, attention should be once again directed to the mentioned earlier gene-gene interaction of* LIG3* with the 589Lys/Glu polymorphism of the* EXO1* gene. Furthermore, not only does the potential protective effect of the Arg allele of the 242Pro/Arg* PolB* SNP and the increased CRC risk associated with the 780Arg/His SNP of* LIG3* and 589Lys/Glu SNP of* EXO1* merit attention, but also, more importantly, the modulation of risk induced by gene-gene interactions identified in this study can significantly affect individual predisposition to the development of cancer.

## 5. Conclusions

Decreased BER activity may play a crucial role in the pathogenesis, development, and progression of colorectal cancer, as the activity of BER is distinctly reduced in lymphocytes and cancer tissue from CRC individuals. In addition, the genotyping of SNPs which have so far not been thought to be associated with CRC (*LIG3*,* PolB*, and* EXO1*) suggests that potential BER dysfunction may lay not only in its first steps, but equally or at even greater level in the gap-filling events. We believe that our results are promising, yet further studies are needed on this subject to establish a link between a given polymorphism and its phenotypic effect in the modulation of BER activity and thus its impact on carcinogenesis.

## Figures and Tables

**Table 1:** The refSNP and thermal conditions used in the PCR reaction.

Gene	*PolB *	*LIG3 *	*EXO1 *
Polymorphism	242Pro/Arg	780Arg/His	589Lys/Glu

refSNP	3136797	3136025	1047840

Thermal conditions	(1) 95°C—10 min		
	(2) 92°C—15 sec		
	(3) 60°C—1 min		
	(4) Step 2 and 3—45x		

Dyes	ROX, HEX, and FAM		

Ref. dye	ROX		

**Table 2:** The distribution of genotypes, allele frequencies, and the analysis of the odds ratio (OR) for 589Lys/Glu polymorphism of *EXO1* gene in patients with colorectal cancer (CRC) and the control group.

Genotype/allele	Patients *n* = 309	Controls *n* = 304^*∗*^	OR (95% CI)	*p*
Lys/Lys	57	69	1 (ref.)	—
Lys/Glu	**203**	**147**	**1.672 (1.109**–**2.519)**	**0.014**
Glu/Glu	49	88	0.674 (0.411–1.106)	0.118
Lys	317	285	1 (ref.)	—
Glu	301	323	0.838 (0.670–1.048)	0.121

^*∗*^Genotype distribution in Hardy-Weinberg equilibrium; *χ* ^2^ = 0.612.

**Table 3:** The distribution of genotypes, allele frequencies, and the analysis of the odds ratio (OR) for 242Pro/Arg polymorphism of *PolB* gene in patients with colorectal cancer (CRC) and the control group.

Genotype/allele	Patients *n* = 303	Controls *n* = 302^*∗*^	OR (95% CI)	*p*
Pro/Pro	147	121	1 (ref.)	—
Pro/Arg	123	142	0.713 (0.507–1.003)	0.052
Arg/Arg	33	39	0.697 (0.413–1.174)	0.174
Pro	417	384	1 (ref.)	—
Arg	**189**	**220**	**0.772 (0.601**–**0.994)**	**0.044**

^*∗*^Genotype distribution in Hardy-Weinberg equilibrium; *χ* ^2^ = 0.791.

**Table 4:** The distribution of genotypes, allele frequencies, and the analysis of the odds ratio (OR) for 780Arg/His polymorphism of *LIG3* gene in patients with colorectal cancer (CRC) and the control group.

Genotype/allele	Patients *n* = 310	Controls *n* = 305^*∗*^	OR (95% CI)	*p*
Arg/Arg	101	121	1 (ref.)	—
Arg/His	**173**	**132**	**1.570 (1.109**–**2.224)**	**0.011**
His/His	36	52	0.829 (0.503–1.368)	0.462
Arg	375	374	1 (ref.)	—
His	245	236	1.035 (0.823–1.302)	0.764

^*∗*^Genotype distribution in Hardy-Weinberg equilibrium; *χ* ^2^ = 0.125.

**Table 5:** The distribution of genotypes and the analysis of the odds ratio (OR) for gene-gene interactions: 589Lys/Glu *EXO1* and 242Pro/Arg *PolB *in patients with colorectal cancer (CRC) and the control group.

Genotype	Patients *n* = 302	Controls *n* = 302	OR (95% CI)	*p*
Lys/Lys-Pro/Pro	24	28	1 (ref.)	—
Lys/Lys-Pro/Arg	20	26	0.897 (0.404–1.994)	0.791
Lys/Lys-Arg/Arg	11	15	0.856 (0.331–2.212)	0.752
Lys/Glu-Pro/Pro	**99**	**51**	**2.265 (1.193–4.301)**	**0.011**
Lys/Glu-Pro/Arg	81	72	1.313 (0.698–2.467)	0.396
Lys/Glu-Arg/Arg	19	23	0.964 (0.426–2.180)	0.920
Glu/Glu-Pro/Pro	23	42	0.639 (0.303–1.346)	0.238
Glu/Glu-Pro/Arg	22	44	0.583 (0.276–1.232)	0.156
Glu/Glu-Arg/Arg	3	1	—	—

**Table 6:** The distribution of genotypes and the analysis of the odds ratio (OR) for gene-gene interactions: 589Lys/Glu *EXO1* and 780Arg/His *LIG3 *in patients with colorectal cancer (CRC) and the control group.

Genotype	Patients *n* = 302	Controls *n* = 302	OR (95% CI)	*p*
Lys/Lys-Arg/Arg	21	27	1 (ref.)	—
Lys/Lys-Arg/His	31	27	1.476 (0.684–3.185)	0.320
Lys/Lys-His/His	3	15	—	—
Lys/Glu-Arg/Arg	64	46	1.789 (0.902–3.547)	0.094
Lys/Glu-Arg/His	**118**	**77**	**1.970 (1.041–3.731)**	**0.036**
Lys/Glu-His/His	17	23	0.950 (0.407–2.218)	0.920
Glu/Glu-Arg/Arg	**15**	**48**	**0.402 (0.178–0.906)**	**0.026**
Glu/Glu-Arg/His	18	26	0.890 (0.389–2.038)	0.777
Glu/Glu-His/His	15	13	1.484 (0.582–3.784)	0.409

**Table 7:** The distribution of genotypes and the analysis of the odds ratio (OR) for gene-gene interactions: 242Pro/Arg *PolB* and 780Arg/His *LIG3 *in patients with colorectal cancer (CRC) and the control group.

Genotype	Patients *n* = 302	Controls *n* = 302	OR (95% CI)	*p*
Pro/Pro-Arg/Arg	52	56	1 (ref.)	—
Pro/Pro-Arg/His	**82**	**41**	**2.154 (1.265–3.667)**	**0.004**
Pro/Pro-His/His	12	24	0.539 (0.245–1.185)	0.121
Pro/Arg-Arg/Arg	38	48	0.853 (0.483–1.506)	0.584
Pro/Arg-Arg/His	66	73	0.974 (0.589–1.611)	0.920
Pro/Arg-His/His	19	21	0.974 (0.471–2.015)	1.000
Arg/Arg-Arg/Arg	9	15	0.646 (0.261–1.603)	0.343
Arg/Arg-Arg/His	20	17	1.267 (0.599–2.679)	0.538
Arg/Arg-His/His	4	7	—	—
